# Ao38, a new cell line from eggs of the black witch moth, *Ascalapha odorata *(Lepidoptera: Noctuidae), is permissive for AcMNPV infection and produces high levels of recombinant proteins

**DOI:** 10.1186/1472-6750-10-50

**Published:** 2010-07-05

**Authors:** Yoshifumi Hashimoto, Sheng Zhang, Gary W Blissard

**Affiliations:** 1 Boyce Thompson Institute at Cornell University, Tower Road, Ithaca NY 14853 USA; 2 Proteomics and Mass Spectrometry Facility, Cornell University, Tower Road, Ithaca NY 14853 USA

## Abstract

**Background:**

The insect cell line is a critical component in the production of recombinant proteins in the baculovirus expression system and new cell lines hold the promise of increasing both quantity and quality of protein production.

**Results:**

Seventy cell lines were established by single-cell cloning from a primary culture of cells derived from eggs of the black witch moth (*Ascalapha odorata*; Lepidoptera, Noctuidae). Among 8 rapidly growing lines, cell line 38 (Ao38) was selected for further analysis, based on susceptibility to AcMNPV infection and production of secreted alkaline phosphatase (SEAP) from a baculovirus expression vector. In comparisons with low-passage High Five (BTI-Tn-5B1-4) cells, infected Ao38 cells produced β-galactosidase and SEAP at levels higher (153% and 150%, respectively) than those measured from High Five cells. Analysis of N-glycans of SEAP produced in Ao38 cells revealed two N-glycosylation sites and glycosylation patterns similar to those reported for High Five and Sf9 cells. Glycopeptide isoforms consisted of pauci- or oligomannose, with and without fucose on N-acetylglucosamine(s) linked to asparagine residues. Estimates of Ao38 cell volume suggest that Ao38 cells are approximately 2.5× larger than Sf9 cells but only approximately 74% of the size of High Five cells. Ao38 cells were highly susceptible to AcMNPV infection, similar to infectivity of Sf9 cells. Production of infectious AcMNPV budded virions from Ao38 cells peaked at approximately 4.5 × 10^7 ^IU/ml, exceeding that from High Five cells while lower than that from Sf9 cells. Ao38 cells grew rapidly in stationary culture with a population doubling time of 20.2 hr, and Ao38 cells were readily adapted to serum-free medium (Sf-900III) and to a suspension culture system. Analysis of Ao38 and a parental *Ascalapha odorata *cell line indicated that these lines were free of the alphanodavirus that was recently identified as an adventitious agent in High Five cell lines.

**Conclusions:**

Ao38 cells represent a highly productive new insect cell line that will be useful for heterologous protein expression and other applications in biotechnology.

## Background

Insect cell lines are essential for basic studies of insect viruses and arboviruses and represent critical components in the baculovirus expression vector system. In addition insect cell lines have been used for studies of immunological, toxicological, and hormonal responses [1]. For instance, hemocyte-like cell lines have been used to characterize signaling pathways and other processes regulating hemocyte immune responses [2,3] and an epidermal cell line derived from the integument of *Helicoverpa armigera *showed gene expression responses to 20-hydroxyecdysone [4]. An insect specific scorpion toxin, AaIT, was demonstrated to be highly toxic to Sf9 cells but not to a human MCF-7 cells [5]. New cell lines that are permissive for replication of the *Autographa californica *nucleopolyhedrovirus (AcMNPV) have potential for use in biotechnological applications related to the baculovirus expression vector (BEV) system. Specifically, cell lines that provide improved protein production or post-translational processing are particularly desirable as they have the promise of higher protein yields, and may provide more biologically active or useful recombinant proteins for research, therapeutics, or vaccine production. For applications in biotechnology, desirable characteristics of an insect cell line include rapid growth (cell division times of ≤ 24 h), adaptation to and rapid growth in large-scale suspension cultures, growth in serum-free media, and susceptibility to infection by AcMNPV and/or other expression vectors. An additional factor in the utility of an insect cell line is the possible presence of adventitious agents. In some cases, the presence of such agents may pose no difficulties. In other cases, cell lines that are free of adventitious agents are more highly desirable since industrial production of human therapeutic proteins or vaccine candidates must adhere to rigorous specifications related to purity and content.

The baculovirus AcMNPV is one of the most commonly used and well-developed of eukaryotic protein expression vectors. This is due in large part to the so-called hyper expression of genes inserted under the control of the polyhedrin promoter of this virus. While very late genes of AcMNPV are expressed at extremely high levels, the level of expression achieved from heterologous proteins may vary considerably in different cell lines. Lines such as the High Five (BTI-Tn-5B1-4) cell line have been used widely since they were initially shown to produce as much as 7 fold higher levels of heterologous protein when compared with standard Sf9 cell lines [6]. While the High Five cell line likely represents the most highly productive cell line currently in widespread use, there remains enormous potential for development of new cell lines from different insect species, from different tissue sources, and by engineering existing cell lines for specific improvements.

In the current studies, we generated primary cell cultures and individual cell lines from eggs of the black witch moth (*Ascalapha odorata*; Lepidoptera, Noctuidae), a large moth indigenous to Central and North America. Here we describe the generation and analysis of single-cell derived lines from primary cultures, and the characterization of one line, Ao38. To examine the potential utility of Ao38 cells, we measured the production of secreted human placental alkaline phosphatase (SEAP) and β-galactosidase expressed from recombinant AcMNPV baculoviruses. We also examined cell growth under various conditions and found that Ao38 cells adapted readily to serum-free culture medium and to a suspension culture system. We also found that Ao38 cells appear to be free of an alphanodavirus that was recently reported to latently or persistently infect High Five cells.

## Results

### Primary *Ascalapha odorata *cell cultures and cell line cloning

**Figure 1:**
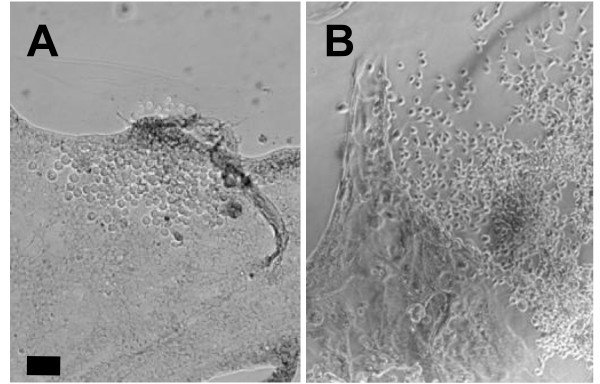
**Generation of *Ascalapha odorata *cell lines**. Cell line development from a primary culture of *Ascalapha odorata *cells. Seventy-seven days after initiating primary cultures from *A. odorata *eggs, the Ao 2.0AA primary culture showed three cell foci containing proliferating cells. Left and right panels show Foci 1 and 2, respectively, which generated cells with distinct morphologies. The black bar represents 100 μm.

**Figure 2:**
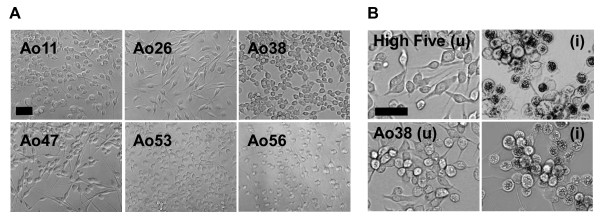
**Clonal *Ascalapha odorata *cell lines and AcMNPV infection**. (A) Clonal cell lines Ao11, Ao26, Ao38, Ao47, Ao53, and Ao56, are shown in the panels. The black bar represents 50 μm. (B) Comparison of uninfected and infected High Five and Ao38 cell lines. Left panels show uninfected High Five cells and Ao38 cells. Right panels show corresponding AcMNPV-infected High Five and Ao38 cells at 48 h pi. The black bar represents 50 μm.

Primary cultures of cells were generated from *Ascalapha odorata *egg tissue suspensions in various dilutions of TNMFH medium. Floating and attached cells were separated and maintained separately. Seventy-seven days after primary cultures were initiated, several isolated colonies of cells were observed in culture flasks containing attached cells (cultures 1.0AA, 2.0AA, and 3.0AA) although vigorous cell proliferation occurred only in culture 2.0AA which contained three distinct foci of cells producing proliferating cells at the edge of dark brown flat tissue explants. Two foci producing cells with different shapes and sizes are shown in Fig. 1. Minor foci producing proliferating cells were also present in culture 2.0AA. The parental culture consisting of mixed cells derived from 2.0AA was named AoP (*Ascalapha odorata *Parental) and was first sub-cultured 92 days after the primary cell culture was initiated. AoP was initially passed at 4 - 5 day intervals (by diluting cells 1:10 in fresh TNMFH medium) and later by similar dilution at 3-day intervals. Single cell-derived lines were generated by dilution of cells from the 2.0AA primary culture in 96-well plates, and single cells were confirmed by visual screening. Twenty-three rapidly growing cell lines were obtained from 70 single cell-derived Ao cell lines generated in this manner. Some of the rapidly growing cell lines showed variation in morphology, size, and aggregation (Fig. 2A).

### Susceptibility to AcMNPV infection

In preliminary studies, AoP and AoF1-F3 cells were challenged with WT AcMNPV at 1 IU/cell. AcMNPV virus infection and replication were observed in all cultures. Rapidly growing Ao cell lines (Ao11, Ao24, Ao26, Ao38, Ao45, and Ao56) were initially screened by analyzing protein production from cell lines infected with a recombinant AcMNPV (SEAP-AcMNPV) that expresses secreted alkaline phosphatase (SEAP) [7]. Based on that analysis, cell line Ao38 (a single cell-derived line from AoP) was selected for further studies. A recombinant AcMNPV expressing β-galactosidase (βgal-AcMNPV) was also used to evaluate the Ao38 cell line for susceptibility to viral infection and for analysis of recombinant protein production [6] (see below).

### Morphology and growth characteristics

Similar to the morphology of High Five cells, Ao38 cells have a flat spindle-like shape in TNMFH medium (Figs. 2A and 2B). Based on microscopic examination, the average cell size of Ao38 cells appeared similar to that of High Five cells although the general morphology shows slight differences in cell shape, with High Five cells exhibiting slightly longer cell extensions. Some cell aggregates were observed in Ao38 cell culture as cell densities approached confluency and this was observed in both TNMFH and Sf-900III media. Aggregates of cells appeared to consist of approximately 50 to 100 cells.

### Adaptation to serum-free medium

Adaptation of Ao38 cells from TNMFH medium into serum free medium (Sf-900III) was performed in two steps, by incrementally lowering the FBS concentration over a 12 day period. Thereafter, the culture was passed by diluting cells 1:10 in fresh medium. The interval between the passages decreased from 8 to 3 days over time (approximately < 3 weeks). During adaptation to Sf-900III medium, the cell morphology changed slightly, becoming more rounded. After adapting Ao38 cells to Sf-900III medium, we also attempted to adapt Ao38 cells to several other commercially available serum-free media formulations, including Sf-900II, Ex-Cell 420, Express V, and ESF921. Ao38 cells cultured in Sf-900II and Ex-Cell 420 media were maintained for several passages without apparent ill effects. However, in those media the cells later began dividing more slowly and showing irregular shapes and sizes, and granules were observed in the cytoplasm. Express V and ESF921 media did not initially support growth of Sf-900III-adapted Ao38 cells (data not shown) and extended efforts were not attempted.

### Adaptation to suspension culture

**Figure 3:**
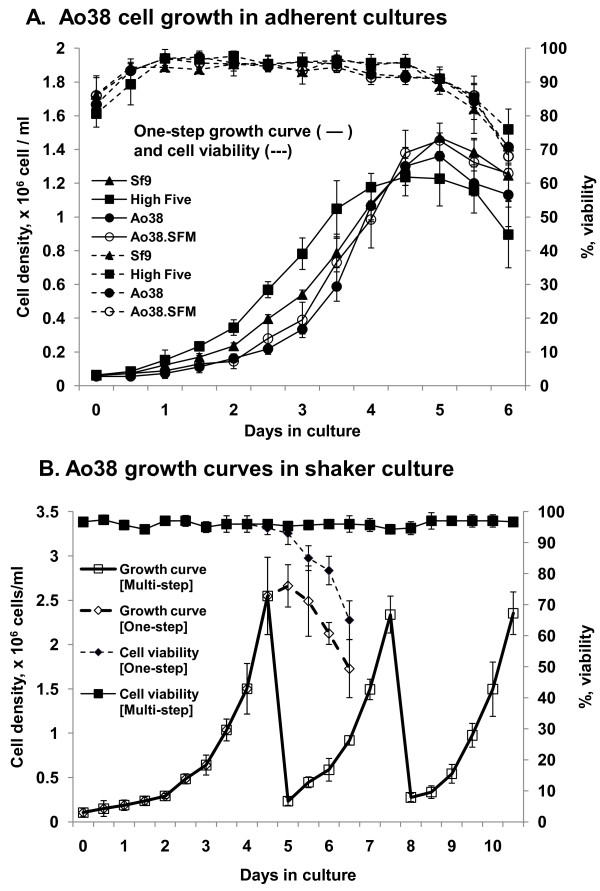
**Growth of Ao38 cells in culture**. (A) The graph shows a one-step cell culture growth curve (solid line) of adherent cells. In addition, measurements of cell viability (dashed lines) are also included. Cell lines Sf9 (closed triangle), High Five (closed squares), and Ao38 (closed circles) grown in TNMFH medium were also compared with Ao38 cells grown in Sf-900III medium (open circles). All data points represent averages of triplicate samples and standard deviations are indicated by bars. (B) The graph shows a one step (dashed line) and a multi-step step (solid line) growth curve (open boxes) and viability (closed boxes) of suspension-culture Ao38 cells that were grown in Sf-900III medium in a shaker culture vessel at 100 rpm. Cells were diluted to approximately 0.25 × 10^6 ^cells/ml in fresh media on days 5 and 8. Data points represent the average of triplicate experiments and standard deviations are plotted.

After > 50 passages of Ao38 cells in Sf-900III medium in T-flasks, the cells were used to establish a suspension (shaker) culture. Approximately 2 × 10^5 ^cells/ml (in 80 ml of Sf-900III medium) were added to a 250 ml Erlenmeyer flask (Corning, Cat. No. 431046) and placed on a shaker platform at 100 rpm. After growth for approximately 3-4 days in shaker cultures, cell aggregates were removed by placing the flask on a stationary platform and allowing aggregates to settle for 3-5 min. The single and less aggregated cells from the top of the culture flask were then removed to another culture flask and placed again at 100 rpm. After repeating this process for five passages, the majority of cells in the shaker culture appeared to be single suspended cells and cell morphology did not appear to change during adaptation to the shaker culture system. Passage of cells by diluting 1:10 in fresh medium every 3 days appeared to be optimum for maintaining shaker cultures of Ao38 cells (Fig. 3B).

### Cell Growth in adherent and suspension cultures

Growth rates for attached Ao38 cells grown in T-flasks were determined by one-step growth curves. For these studies Ao38 cells were propagated in either TNMFH or Sf-900III culture media and for comparisons, High Five and Sf9 cells were propagated in TNMFH culture medium (Fig. 3A). During the first four days, Sf9 and High Five cells grew at a higher rate than that of Ao38 cells. However, High Five cells peaked at approximately 1.2 × 10^6 ^cells/ml at approximately 4.5 days whereas Ao38 and Sf9 cell densities peaked slightly later, at 5 days, with densities of 1.36 - 1.47 × 10^6 ^cells/ml. Doubling times for Sf9, High Five, Ao38 (in TNMFH) and Ao38 (in Sf-900III) cells were 22.6, 21.4, 20.2, and 20.9 hr, respectively. Thus growth rates and doubling times were similar for Ao38, High Five, and Sf9 cells. In addition, growth curves of Ao38 cells in serum-containing and serum-free media were similar to those of High Five and Sf9 cells in TNMFH. While the above data represent studies in adherent cultures, we also examined Ao38 cell growth in suspension cultures (Fig. 3B). Growth curves and cell viability assays in both one-step and continuous multi-step shaker cultures showed greater than 90% cell viability and cell densities up to 2.5 × 10^6 ^cells/ml.

### Cell volume comparisons

**Figure 4:**
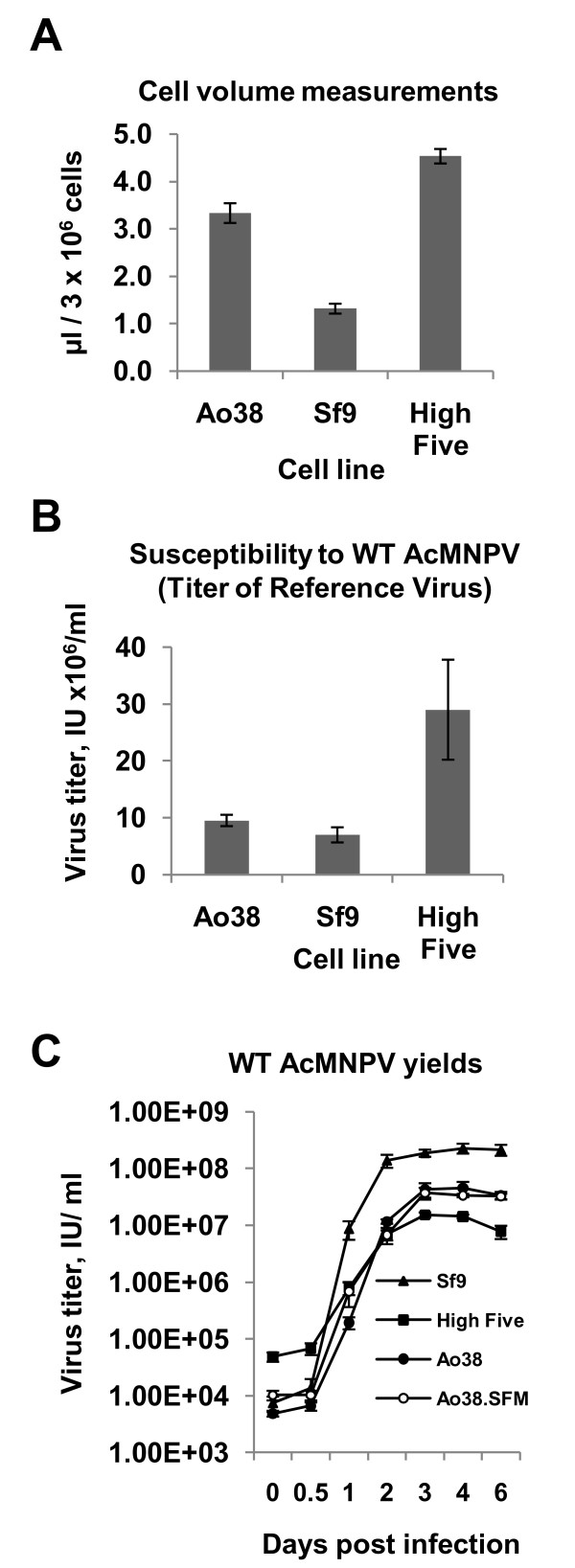
**Ao38 cell volumes, AcMNPV susceptibility, and virus yields**. (A) Measurements of packed cell volumes (PCV, μl per 3 × 10^6 ^cells) of Ao38, Sf9 and High Five cells are compared in a bar graph. Gray bars represent averages and standard deviations from 3 replicate measurements are indicated. (B) Susceptibility of Ao38, Sf9, and High Five cells to AcMNPV infection. The relative susceptibilities of Ao38, Sf9, and High Five cells to AcMNPV infection was measured by infecting each cell line with an AcMNPV virus (βgal-AcMNPV, 1 × 10^7 ^IU/ml) that was initially titred on Sf9 cells. The same virus preparation (βgal-AcMNPV) was then titred on each cell line. Gray bars represent averages of virus titers determined on each cell line at 4 days p.i. and standard deviations of triplicate infections are indicated. (C) Comparisons of WT AcMNPV production from Sf9, High Five, and Ao38 cells in TNMFH medium, and from Ao38 cells in Sf-900III serum-free medium. Virus titres were determined from the supernatants of triplicate infections of each cell line with WT AcMNPV at various times post infection (Y-axis).

To compare the relative size of Ao38 cells with that of the commonly used Sf9 and High Five cells, we measured packed cell volumes (PCV) of cells from each cell line [8]. PCV measurements suggest that the size of Ao38 cells is 2.5 and 0.7 times that of Sf9 and High Five cells, respectively (Fig. 4A). Thus, Ao38 cells were substantially larger than Sf9 cells yet appear to be slightly smaller than High Five cells (which are approximately 3 times the volume of Sf9 cells).

### Virus susceptibility

To examine the relative susceptibility of different cell lines to infection by AcMNPV, we used a standard preparation of the βgal-AcMNPV virus that was previously titred on Sf9 cells (1 × 10^7 ^IU/ml), for infection of each cell line. In parallel, the titre of the standard virus preparation was determined on each candidate cell line. Titration of the standard virus inoculum on Sf9 cells produced a titer of 0.70 (+/- 0.13) × 10^7 ^IU/ml. The titres determined on Ao38 and reference High Five cells were 0.95 (+/- 0.10) × 10^7 ^and 2.90 (+/- 0.88) × 10^7 ^IU/ml, respectively. These results indicated that Ao38 cells were similar to, or slightly more susceptible to AcMNPV infection than Sf9 cells. In addition and as reported previously [9], High Five cells were approximately three times more susceptible to AcMNPV infection than Sf9 cells (Fig. 4B).

### AcMNPV replication in Ao38 cells

To compare AcMNPV production in Ao38 cells to that in Sf9 and High Five cell lines, we infected cells with WT AcMNPV and generated one-step viral growth curves in each cell line (Fig. 4C). Peak virus titres were 1.5 × 10^7^, 4.5 × 10^7^, and 2.8 × 10^8 ^IU/ml in High Five, Ao38, and Sf9 cell lines, respectively. Although the kinetics of viral production were generally similar, AcMNPV titres from Sf9 cells appeared to peak earlier that in Ao38 or High Five cells. Thus, Ao38 cells generated virus titers at relatively high levels (4.5 × 10^7 ^IU/ml) that exceeded levels measured from High Five cells, but below that from Sf9 cells. We also examined Ao38 cells that were propagated in, and infected in serum-containing TNMFH and in serum-free (Sf-900III) media. Cells propagated and infected in both media resulted in similar AcMNPV yields. Thus the presence of serum did not appear to substantially affect virus production.

### Recombinant protein production in Ao38 cells

**Figure 5:**
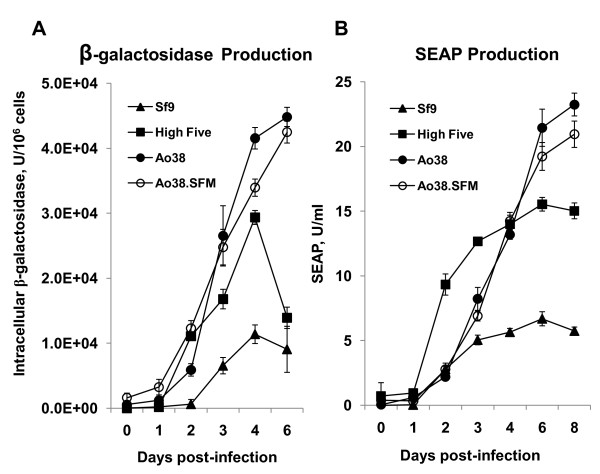
**β-gal and SEAP production of Sf9, High Five, and Ao38 cells**. (A) Beta-galactosidase production was measured from Ao38, High Five, and Sf9 cells at various time post infection (Days post infection) by virus βgal-AcMNPV. Ao38.SFM represents Ao38 cells grown in serum-free medium (Sf-900III medium) and all other samples were produced in TNMFH containing 10% FBS. Data points and bars represent averages and standard deviations, respectively, from triplicate infections. (B) Secreted human placental alkaline phosphatase (SEAP) was measured from Ao38, High Five, and Sf9 cells at various time post infection (Days post infection) by virus SEAP-AcMNPV. Ao38.SFM represents Ao38 cells grown in serum-free medium (Sf-900III medium) and all other samples were produced in TNMFH containing 10% FBS. Data points and bars represent averages and standard deviations, respectively, from triplicate infections.

For maximal utility of new lepidopteran insect cell lines in biotechnology, the capacity for high-level protein production is an important characteristic. To determine whether Ao38 cells were suitable for high-level protein production, Ao38 cells propagated in both TNMFH and Sf-900III media were infected with recombinant AcMNPV viruses that express either an intracellular, or a secreted reporter protein under the control of the AcMNPV polyhedrin promoter. For these studies, Ao38 cells were compared with Sf9 and High Five cells as standards for high-level protein production. The virus βgal-AcMNPV was used for studies of intracellular protein production, and recombinant β-galactosidase expressed in the cell lines was quantified (see Materials and Methods). β-galactosidase production peaked at day 4 for High Five and Sf9 cells, and at day 6 for Ao38 cells. Peak levels for High Five cells were 29.4 × 10^3 ^U/10^6 ^cells whereas those for Ao38 were 44.8 × 10^3 ^U/10^6 ^cells (Fig. 5A). Thus, while β-galactosidase production peaked one day later in Ao38 cells, expression levels were 152% of that observed for High Five cells and 231% of that observed for Sf9 cells.

To examine the production of a secreted protein in Ao38 cells, cells were infected with SEAP-AcMNPV (MOI 10) and levels of secreted alkaline phosphatase (SEAP) were measured at various times post infection. SEAP production from infected Sf9 and High Five cells was examined in parallel. SEAP production in Ao38 cells was measured at levels of > 20 U/ml (Fig. 5B). As also observed for β-galactosidase production, SEAP production from infected Ao38 cells continued for a longer period of time and was 300% of that observed from Sf9 cells and 150% of that from High Five cells. Thus, in both cases of intracellular and secreted proteins, recombinant protein production in Ao38 cells peaked slightly later and was substantially higher than that from High Five or Sf9 cells.

### Analysis of SEAP N-glycans

**Figure 6:**
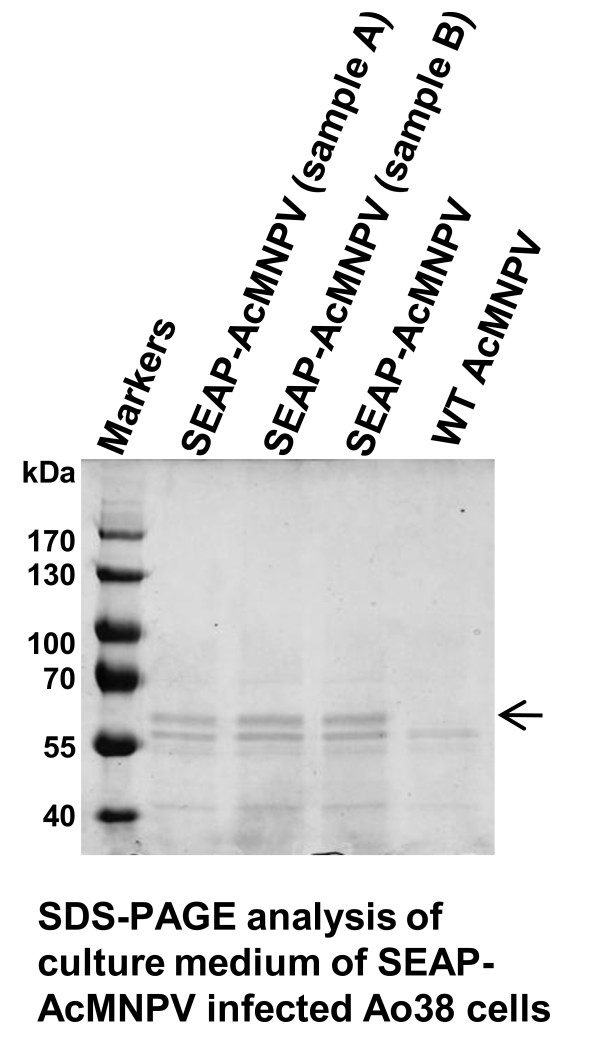
**SDS-PAGE analysis of SEAP production in SEAP-AcMNPV infected Ao38 cells**. Samples of 20 μl from the supernatant of SEAP-AcMNPV infected Ao38 cells grown in Sf-900III medium were electrophoresed and major protein bands identified by Coomassie blue staining. Lane 1; protein marker sizes in kDa are indicated on the left. Lanes 2-3 represent two independent preparations of SEAP-AcMNPV infected culture medium (20 μl). Lane 4, SEAP-AcMNPV infected culture medium (20 μl) after centrifugation (80,520 × g for 60 min) to remove baculovirus virions. Lane 5, Control from WT AcMNPV infected culture medium (20 μl). An arrow indicates the position of the protein band that was excised and subjected to N-glycan analysis by LC-MS/MS.

To examine protein glycosylation in Ao38 cells, we used LC-MS/MS to examine the N-linked carbohydrate from the 64 kDa SEAP glycoprotein produced from the recombinant virus SEAP-AcMNPV. Using SDS-PAGE analysis of culture medium from SEAP-AcMNPV infected Ao38 cells, we identified an intensely staining band of approximately 64 kDa (Fig. 6). A similar band was absent from culture medium from WT AcMNPV infected Ao38 cells. The 64 kDa band was excised and the tryptic peptide composition was examined by LC analysis and confirmed as SEAP. The SEAP protein has two N-glycosylation motifs (N-X-S/T/C, where X can be any amino acid except proline) located at N139 and N266. The two predicted motifs are located in the tryptic glycopeptides, FNQCN^139^TTR and YVWN^266^R. The observed masses of these glycopeptides are summarized in Additional file 1. The Additional file 1 also shows the proposed structures of these N-glycans (based on theoretical sugar masses) and their predicted relative abundance. The mass spectrometry spectra showed the presence of an isoform at the N^139 ^glycopeptide with a difference in mass of 14 Da in the amino acid backbone. MS/MS spectra showed the additional 14 Da on the alkylated cysteine residue 138 (data not shown). We suspect that the difference reflects an additional methylene (-CH_2_-) group and is caused by residual acrylamide in SDS gel labeling free cysteine to produce propinamide-Cys rather than expected acetamide-Cys or the presence of a mixture of cysteine and homo-cysteine in the tryptic peptide. In both N-glycans at N^139^, the major sugar composition of the N-glycan was a fucosylated, mono- or bi-antennary, pauci- or oligo-mannose structure [Man2-4GlcNAc2Fuc1,2]. The N-glycan at N^266 ^had a molecular mass corresponding to a non-fucosylated pauci- or oligo-mannose structure [Man2-6GalNAc2]. A small portion of the N-glycan corresponds to a bi-antennary tri-mannose structure with a GlcNAc at one branch [GlcNAcMan3GlcNAc2]. In Sf9 cells infected with SEAP-AcMNPV, pauci- or oligo-mannnocidic N-glycans with and without fucose were also produced in the major portion of N-glycan. In High Five cells infected with SEAP-AcMNPV, similar glycan components were also found by PNGase F digestion followed by CE capillary analysis of released glycans [10]. But complex type N-glycans such as Gal3GlucNAc3Man3GucNAc2, Gal3GlucNAc3Man3GucNAc2Fuc, Gal3GlucNAc4Man3GucNAc2Fuc were reported as a substantial proportion (>40%) of the total N-glycans in High Five cells [11]. SEAP produced from CHO cells contains complex and sialylated N-glycan structures in >50% of the N-glycans [12]. A comparison of biochemical features of SEAPs expressed from three insect cell lines is summarized in Table 1.

**Table 1:** Comparison of expression levels and N-glycan composition of SEAP in Sf9, High Five, and Ao38 cells

Cell line	Sf9	High Five	Ao38
Expression level (U/ml)	6	11.6	23.2

Identified glycans	Man2, Man3, Man3F, Man4, Man5-8	Man2, Man3, Man4, Man4F, Man5-8	Man2F, Man3F, Man4F, Man3F1,2
			Man2-6, HexNAc-Man3

	Man3F: 30%	Man3: 25%	Man3F: 53% for N^139^
Major component^a^	Man4: 40%	Man4F: 15%	Man6: 31% for N^266^
			Man3: 29% for N^266^

^a ^Percent composition of the major component of N-glycans were determined by LC/MS/MS for each glycan (N139 or N266) of Ao38-expressed SEAP, and compared with published data from total glycans of SEAP expressed in Sf9 and High Five cells (see reference [10]).

### Absence of TNCL Virus

**Figure 7:**
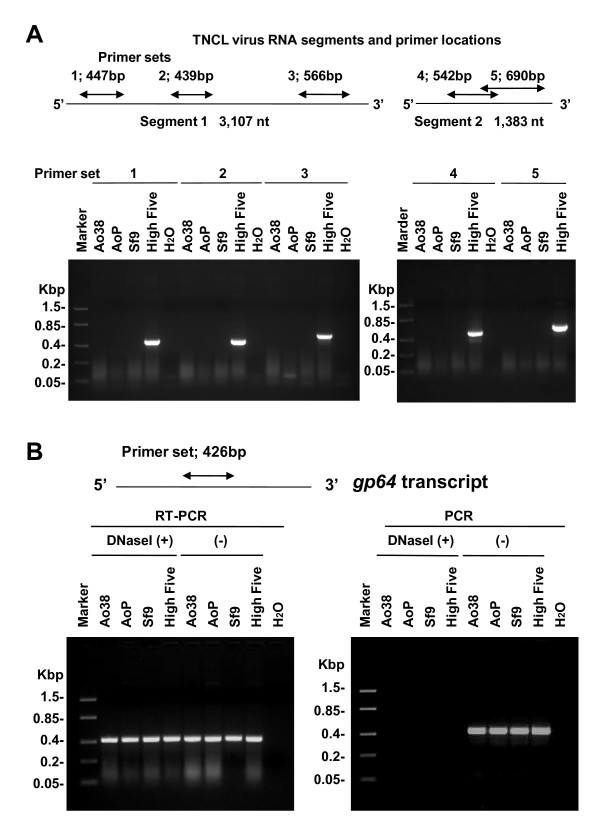
**RT-PCR analysis and TNCL virus detection**. (A) To examine cell lines for the presence of a TNCL virus, five primer pairs were used for RT-PCR analysis of total RNA from cell lines. Ao38, AoP (passage 5), Sf9, and High Five cells were infected with AcMNPV 48 h prior to RNA isolation. The relative locations of primer pairs on TNCL Virus RNAs 1 and 2 are indicated in the upper diagram, and primer sequences are listed in Table 2. Primer sets were labeled as follows: Set 1, Noda-R1-190F and Noda-R1-636R; Set 2, Noda-R1-1093F and Noda-R1-1531R, Set 3; Noda-R1-2368F and Noda-R1-2933R; Set 4, Noda-R2-269F and Noda-R2-810R; Set 5, Noda-D4 and Noda-U4. Primer sets and cell lines are indicated above each lane, and sizes (Kbp) of marker DNAs are indicated on the left of each gel. (B). RT-PCR analysis of gp64 transcript RNA control. RT-PCR (left) and PCR (right) were used to analyze total RNA from Ao38, Ao-P (passage 5), Sf9, and High Five cells infected with WT AcMNPV, AcMNPV gp64 primers are listed in Table 2. Samples treated with DNaseI (+) or not treated with DNaseI (-) are indicated above lanes. Sizes (kbp) of marker DNAs are indicated on the left.

Recently, an alphanodavirus named Tn5 Cell Line Virus, or TNCL Virus, was described in High Five cells (BTI-Tn-5B1-4). TNCL virus was induced upon infection by WT and recombinant AcMNPV [13]. In order to determine a) if that virus resulted from the initial cell cultures or isolation of BTI-Tn-5B1-4 cells, and b) whether Ao38 or other Ao cell lines also carried this virus, we used RT-PCR to examine AcMNPV infected and uninfected cells from Ao38, Sf9, and High Five. For analysis of High Five cells, we used a relatively early passage of that cell line (passage 98). RT-PCR was performed to detect TNCL virus RNAs in total RNA isolated from WT AcMNPV infected AoP (passage 5) and Ao38 cells (Fig. 7A). Five primer sets were used to sample the bipartite RNA genome of TNCL virus (Table 2, Fig. 7A). The five primer sets produced amplicons with the expected sizes from AcMNPV-infected High Five cell RNA, confirming the presence of the TNCL virus. However, in a parallel analysis no amplicons were detected from cellular RNA preparations from AcMNPV-infected AoP, Ao38 or Sf9 cells. As an internal control, AcMNPV *gp64 *transcripts were examined in each RNA preparation to confirm the quality and specificity of each preparation. One-step RT-PCR and PCR was performed with the RNAs, either with or without DNase I treatment to confirm the detection of transcripts. When a DNase I-treated total RNA preparation from AcMNPV infected cells was used as template, the *gp64*-specific product was detected by one-step RT-PCR, but not by PCR alone, confirming the detection of the gp64 transcript (Fig. 7B).

**Table 2:** Primers used in TNCL virus study

Primer	Sequence (5'-3')	TNCL RNA segment	Position on viral genome*	Direction
Noda-R1-190F	gggaaccgagttacacgcgcattgc	1	190..214	>
Noda-R1-636R	ggtgaatggtgagtcagcatc	1	616..636	<
Noda-R1-1093F	caatctgtcaacgctaggcttatcgg	1	1093..1188	>
Noda-R1-1531R	ccgccctaagttgtagttgttgggacgg	1	1504..1531	<
Noda-R1-2368F	tgtaccgatgcgcttactccgttgatatcgg	1	2368..2398	>
Noda-R1-2933R	ccacgctgggtttctccagcagtgatgttacc	1	2902..2933	<
Noda-R2-269F	ggaatacctgatagatttgaaggcaaag	2	269..296	>
Noda-R2-810R	ggcaatgtttggataccctccaatatgtcg	2	781..810	<
Noda-D4	acatccagatccgatcaagt	2	491..510	>
Noda-U4	gccaggaatgttgcttgcaa	2	1161..1180	<

**Primer**	**Sequence (5'-3')**	***gp64***	Position*	**direction**

C-177Fs	attttggacgctgagggc		109139..109156	<
L-330Rs	cttgttgatgtgcgcatgcatcagctc		108731..108757	>

*; position of each primer is based on sequences of deposited in GenBank under accession numbers EF690537 (TNCL virus RNA1), EF690538 (TNCL virus RNA2), and L22858 (AcMNPV)

## Discussion

In the current studies, we established a variety of proliferating cell lines from *Ascalapha odorata *egg tissue. After generating primary cultures, proliferating cells were identified as individual colonies at approximately 77 and 90 days. Cells from proliferating colonies were used for single cell cloning by limiting dilution at this relatively early stage (13 days after identifying three foci of proliferating cells in culture 2.0AA) in order to preserve the diversity of potentially useful cells. The 23 rapidly growing cell lines exhibited variations in size, shape, substrate adherence and growth rates. In a preliminary screen, cloned cells were examined based on growth characteristics, AcMNPV infectivity, and production of recombinant secreted alkaline phosphatase (SEAP) from a baculovirus vector. From these, one cell line (Ao38) was selected for more detailed analysis.

Ao38 cells grown in TNMFH are moderately-to-tightly adherent, spindle shaped, and are relatively large in comparison with the more commonly used Sf9 cells. Measurements of packed cell volumes indicated that Ao38 cells are larger than Sf9 cells, but smaller than High Five cells. Ao38 cells have a morphology similar to that of High Five cells (Fig. 2B). Our subsequent analysis showed that Ao38 cells had the following culture characteristics. Ao38 cells can be plated at low densities (approximately 5% confluency) without substantial effects on growth rate, and cells grow rapidly (doubling time of approximately 20.2 h) to relatively high densities of over 1.4 × 10^6 ^cells/ml in stationary cultures. As cells grow beyond confluency, some vertical aggregation of cells was observed. The observed clumping may result from a lower level of contact inhibition than observed in other cell lines, and that may contribute to the observation of higher cell densities in adherent and suspension cultures and continued recombinant protein production. Cell clumping was minimized by stepwise adaptation to suspension culture: removing cell aggregates at intervals.

Perhaps the most important and widespread applications of insect cell lines involve the production of secreted proteins that will be later purified from the growth medium. Therefore growth of cells and production of recombinant proteins in serum-free medium is an important characteristic. Ao38 cells were adapted to a commercial serum-free medium, Sf-900III, within a relatively short time period (12 days) and without laborious or stepwise adaptation. However, the Sf-900III-adapted cells did not grow successfully in three other commercial media that have been used for Sf9 and/or High Five cells. Ao38 cells also adapted readily to shaker cultures, using 5 steps to remove aggregates of cells during the first 15 days of growth in the suspension cultures. The procedures for adaptation to suspension cultures were similar to those we have previously used for High Five cells. Cell growth curves in suspension cultures and cell viability assays indicated that Ao38 cells grew optimally in shaker cultures at cell densities ranging from 0.2 × 10^6 ^to 2.5 × 10^6 ^cells per ml.

We also examined Ao38 cells in terms of viral infectivity, yields, and production of recombinant proteins. Comparisons with Sf9 and High Five cells revealed that Ao38 cells were similar to Sf9 cells in susceptibility to WT AcMNPV infection and both were less susceptible than High Five cells. In contrast to viral susceptibility studies, we found that yields of infectious virus were highest in Sf9 cells. WT AcMNPV yields from Ao38 cells were slightly less than observed from Sf9 cells, but higher than virus yields from High Five cells. Thus, in comparisons of viral susceptibility and virus yield in Sf9 and High Five cells, Ao38 cells compare favorably and are intermediate between these two well-established cell lines. The kinetics of β-galactosidase and SEAP protein production also suggest that progression of the infection cycle is similar in the three cell lines although future studies will examine this in more detail. More importantly though, recombinant protein yields from AcMNPV-infected Ao38 cells were higher those from Sf9 and High Five cells. While the kinetics of β-galactosidase production were generally similar among the three cell lines, peak protein levels were substantially higher from Ao38 cells. Although β-galactosidase production in High Five and Sf9 cells appeared to decrease after 4 days, we found that β-galactosidase continued to accumulate through 6 days post infection in Ao38 cells. A decrease in intracellular β-galactosidase activity in Sf9 and High Five cells after 4 or 5 days p.i. has been previously reported [6,14]. Production of peak levels of the secreted protein, SEAP, was most rapid in High Five cells. However, peak SEAP levels in Ao38 cells were approximately 50% greater than peak SEAP levels in High Five cells (Fig. 5B). This may be explained by the observation that SEAP production in Ao38 cells appears to continue over a longer time period, permitting accumulation of higher quantities of the secreted protein. It was previously reported [15] that on a culture volume basis, High Five cells produced at least fivefold more SEAP than Sf9 cells. The maximum concentration of SEAP reported for High Five cells was 9.5 U/ml. These results are comparable to SEAP data from the current study (Fig. 5B). We measured peak SEAP production in High Five cells at 15.5 U/ml whereas peak SEAP production in Ao38 cells was measured at 23.2 U/ml in serum-containing medium, and 20.9 U/ml in serum-free medium. Overall our analysis of protein production using two reporter proteins expressed from recombinant baculoviruses indicates that Ao38 cells are capable of producing higher levels of both intracellular and secreted proteins. The reason for higher level protein production by Ao38 cells is unclear although it may result from either more robust protein synthetic machinery, more efficient protein transport and processing, or some combination of these factors. Alternatively, our preliminary observations suggest that when compared with Sf9 and High Five cells, Ao38 cells appear to remain viable for a longer period after infection initiates, possibly resulting in a prolonged period for protein production. Future studies will address these and additional questions.

Insect cells are known to produce glycoproteins with paucimannnosidic or oligomannose N-glycan structures and this is believed to be due to either divergence of N-glycan processing pathways in subcellular organelles, and/or different regulation of the enzymes responsible for generating complex N-glycan structures [16-18]. To examine and evaluate protein glycosylation in Ao38 cells, we performed LC-MS/MS analysis on the N-glycan of recombinant SEAP protein produced from an AcMNPV expression vector. Based on the consensus sequence for N-linked glycosylation, SEAP has three potential N-glycosylation sites at N^136^, N^139 ^and N^266^, and prior studies suggested that only the N^266 ^site was utilized for glycosylation [12,19]. In the current study of Ao38 cells, we applied the precursor ion scans monitoring the specific marker ion (HexNAc^+ ^at m/z 204) in LC-MS/MS analysis for selective identification of all glycan isoforms associated with each of peptides. We identified N-linked glycosylation of two tryptic peptides at position N^139 ^and N^266 ^which were eluted at 8.5 min and 20 min, respectively under our LC conditions (Additional file 1). This analysis is complementary with previous approaches for released glycans from SEAP protein, allowing us to determine the glycoisoforms and the difference for each of the N-glycan sites. Sugar composition at both N-glycan sites was paucimannosidic or oligomannose, but more mannose residues were present in the N-glycan at N^266^. Interestingly, fucosylation at N-acetylglucosamine linked to Asn selectively occurred for the N-glycan at N^139^. It is possible that the selective fucosylation may have occurred due to the nature of the structure around N^139^. The nature of the fucose linkage (alpha 1,6 or alpha 1,3) in SEAP from Ao38 cells is not known but insect cell lines have been reported to differ in whether or not their glycoproteins contain core alpha (1,3) fucose (reviewed in reference [17]), and fucosylation may have significance in various applications. While recombinant glycoproteins with a core alpha (1,3) fucose may not be problematic as therapeutics [20], they have resulted in false positives when used in diagnostic assays [21]. It will be of interest in future studies to determine whether this specific linkage is found in glycans from Ao38 cells.

Glycans at the N^139 ^residue were predominantly Man3F and Man2F in composition (Additional file 1). In contrast, Man3 and Man6 are the dominant glycan isoforms found at the N^266 ^residue. Nevertheless, the identification of the glycans in SEAP expressed in the Ao38 cell line reported in this work is similar to that identified previously from Sf9 and High Five cell lines [10,11]. It should be noted that the relative quantitation of identified N-linked glycan isoforms should be treated as an estimate since the assumption that all sugar isoforms share a similar ionization efficiency may not be accurate.

Recently an adventitious virus was identified in High Five cell lines. The alphanodavirus, TNCL virus, was identified during production of hepatitis E virus-like particles in High Five cells infected with a recombinant baculovirus vector [13]. Using RT-PCR and primers specific for five regions of the TNCL virus genome, we were unable detect the TNCL Virus in AcMNPV infected Ao38 cells, an uncloned parental cell line (AoP) from *Ascalapha odorata*, or Sf9 cells (a negative control) (Fig. 7A). However, TNCL virus was detected in AcMNPV-infected High Five cells (passage 98) used as a positive control. Persistent viral infections have been observed occasionally in insect cell lines [22,23] and nodaviruses are thought to occasionally infect wild marine invertebrates either latently or persistently [24]. A betanodavirus was also reported to be latently infecting a cell line derived from the brain tissue of the barramundi fish, *Lates calcarifer *[25].

## Conclusions

We have described the development and characterization of a new cell line, Ao38, derived from cells of the Black Witch Moth, *Ascalapha odorata*. Ao38 cells were found to be highly productive for expression of recombinant protein and exceeded protein expression from the well-characterized Sf9 and High Five cell lines. The data described in this study indicate that Ao38 cells will be a valuable new tool for protein expression using the baculovirus expression system, and in addition, Ao38 cells may be important for cell line engineering by insertion of genes into the Ao38 cell genome.

## Methods

### Establishment of cell lines

Primary cultures of cells were prepared on February 19, 2008, from eggs of *Ascalapha odorata *(black witch moth; Lepidoptera, Noctuidae) collected at the Boyce Thompson Institute from adult female moths that were originally collected in Hawaii. In brief, approximately 150 two day old eggs were placed in a 100 μm Nylon cell strainer (BD Falcon) and surface sterilized in the following manner. The eggs were immersed in 2% Clorox for 10 min, rinsed with 70% ethanol for 4 min, then rinsed briefly with autoclaved distilled water. The eggs were then crushed and cells were forced through the nylon mesh strainer with a cell scraper (BD Falcon), into a six well cell culture plate containing 3 ml of TNMFH culture medium [26] supplemented with 10% fetal bovine serum (HyClone, Inc.). The egg cell suspension was diluted to a volume of 10 ml with fresh TNMFH medium. The diluted tissue suspension was then mixed with fresh TNMFH in various ratios, 1:6 to 6:1 (egg cell suspension: TNMFH) to a final volume of 3.5 ml, and each was placed in a 25 cm^2 ^T-flask (Corning). These primary cultures were incubated for one week at 27°C with no change of medium. The primary cultures were passaged by replacing the medium with fresh TNMFH every 2-3 weeks in order to separate floating and attached cells. Floating cells were retained in separate T-flasks. After approximately 11 weeks, three separate cell foci were observed at the periphery of egg tissue explants in a primary culture, 2.0AA. (Culture 2.0AA was derived from an initial mixture of 2.0 ml of egg tissue suspension and 1.5 ml of TNMFH, a 4:3 dilution as described above.) The attached cells of culture 2.0AA were selected by twice removing floating cells. After each cell focus had grown to a few hundred cells, portions of each focus were carefully removed using a micropipette tip and transferred into new T-flasks. Cell lines derived from foci 1 to 3 are referred to as AoF1, AoF2, and AoF3. The parent culture (that contained the remainder of the original 3 foci) was split into two cultures. One was used for single cell cloning by limiting dilution, and the other was used to develop a parental mixed cell line (AoP). AoF1-AoF3 and AoP cells were frozen at passage 1 and 2. Among seventy cell lines cloned as single cells, 23 rapidly growing cell lines were selected for further analysis based on their rapid growth rate in 96 well plates. After amplifying the cells, each cell line was frozen at passage 1 and 2, and used in the current study after over 10 passages in T-flasks. A second primary culture was generated from *A. odorata *eggs and processed in the same manner on March 16, 2009.

### Analysis of cell growth rates

To examine the rate of cell growth, cells (5 × 10^4 ^cells) were seeded in a 6-well cell culture plate and cell density and viability were determined at 12 hour intervals. For density calculations, adherent cells were dislodged from one well of a 6-well plate (9.6 cm^2 ^per well), and suspended in 0.5 ml culture medium. The density and viability of the suspended cells were determined using a hemocytometer and trypan blue staining. A cell growth curve was generated from the results of three independent experiments.

### Cell lines and viruses

Cell lines Sf9 (ATCC No: CRL-1711) [27] and BTI-Tn-5B1-4 (High Five) [7,28] were used as controls in this study. Because protein expression may be reduced in very high passage insect cell lines, we used High Five cells from a relatively low passage stock (84 to 95 passages) for this study. Both cell lines were maintained in TNMFH medium. Wild type (WT) AcMNPV (strain E2) was used in this study. As a reporter for production of intracellular protein, we used a recombinant AcMNPV containing the *Escherichia coli *β-galactosidase gene under the transcriptional control of the polyhedrin gene promoter, a virus that was used in similar studies and previously described [6]. As a reporter for production of secreted protein production, we used a recombinant AcMNPV containing a secreted human placental alkaline phosphatase (SEAP) gene under the control of a polyhedrin promoter [7].

### Cell growth in serum-free medium and suspension culture

To examine Ao38 cell adaptation and growth in serum-free medium, Ao38 cell adaptation was initially examined in several commercial serum-free media formulations. Ao38 cells adapted most rapidly to Sf-900III medium (Invitrogen Corp.), which was used for subsequent studies. For adaptation studies, a fifty percent confluent culture of Ao38 cells in T25 flasks, was first incubated in a mixed medium of TNMFH and Sf-900III (1:9) for 6 days at 27°C. The medium was then changed to 100% Sf-900III, and the culture was incubated for another 6 days. During the later 6-day period, the culture became confluent. The culture was passed by diluting cells 1:10 in Sf-900III medium at 3-4 day intervals, over 10 passages before initiating the cell line characterization studies. Sf-900III-adapted Ao38 cells were also examined for growth in other serum-free media. For those studies, cultures of Sf-900III-adapted Ao38 cells (approximately 80% confluent) were transferred directly into four different media formulations: Sf-900II (Invitrogen), Express V (Invitrogen), ESF921 (Expression Systems), and Ex-Cell 420 (SAFC Biosciences).

Growth of Ao38 cells in suspension cultures was examined in the following manner. Sf-900III-adapted Ao38 cells were initially seeded into a 250 ml tissue culture flask (Corning) by adding 80 ml of Ao38 cells (1 × 10^5 ^cells/ml) in Sf-900III medium. The culture was placed on an orbital shaker at 100 rpm at 27°C. During the first several passages, large cell aggregations of several hundred cells were observed in addition to single cells. To reduce cell aggregation, the culture was removed from the shaker platform and aggregates allowed to settle for 3-5 minutes. The upper phase of the culture (containing single and less aggregated cells) was transferred to another culture flask and returned to the shaker platform as described above. After five repetitions of this process, single cells predominated in the shaker culture. Passage was then carried out by diluting cells 1:10 in fresh medium every three days. Growth curves of cells in the shaker culture were generated by measuring cell density and viability every 12 h. Three replicates of each shaker culture were included in these studies.

### Cell volume comparisons

To compare the relative volumes of Ao38, High Five, and Sf9 cells, we examined the packed-cell volumes (PCV) of each cell line using cells grown in TNMFH medium and VoluPAC tubes (Sartorius). We initially examined the optimum g-force for pelleting cells by comparing PCVs at g-forces of 100, 247, 523, 989, 1490, and 2000 × g for 1 min. At 1,490 and 2,000 × g, consistent PCVs were obtained with each cell line. Therefore, cells (3 × 10^6 ^cells) from a log-phase culture were pipetted into a VoluPAC tube, centrifuged at 2,000 × g for 1 min, and the volume of the cell pellet was measured to determine the PCV. Averages and standard deviations were calculated based on triplicate sample data.

### Comparisons of AcMNPV infectivity and yield

#### Viral infectivity

To compare cell lines for susceptibility to AcMNPV infection, we infected Sf9, High Five, and Ao38 cells in parallel with a recombinant AcMNPV virus that was produced and titred on Sf9 cells. βgal-AcMNPV (1 × 10^7 ^IU/ml, generated and titrated on Sf9 cells) was used to prepare 10^2 ^to 10^9 ^fold virus dilutions. Ten μl of each viral dilution was added to 2 × 10^4 ^Sf9, High Five, or Ao38 cells in 90 μl TNMFH medium in 12 wells of a 96 well cell culture plate. Four days later, the cells were stained with an X-gal solution and incubated at 27°C for 1 hr to identify infected cells and determine viral titres. The experiment was run in triplicate.

#### Virus Yields

To examine and compare AcMNPV virus yields from the Ao38 cell line, three cell lines (Ao38, High Five, and Sf9) were infected with WT AcMNPV. Each of the three cell lines was propagated in TNMFH medium, and in addition Ao38 cells were also propagated in Sf-900III medium. Cells were infected by a 1 h incubation with WT AcMNPV at an MOI of 10 IU/cell, followed by rinsing the cells once with fresh medium. After infection, culture supernatant samples were collected at 24 h or 48 h intervals through 6 days post infection. For each supernatant sample, cells or debris were removed by a low speed centrifugation and the supernatant was used for virus titration by TCID_50 _on Sf9 cells.

### Analysis of recombinant protein production from recombinant AcMNPV

To compare recombinant protein production in Ao38 cells, we infected Ao38, High Five, and Sf9 cells with either a) a recombinant virus (βgal-AcMNPV) expressing the intracellular protein β-galactosidase, or b) a recombinant virus (SEAP-AcMNPV) expressing the secreted protein, SEAP.

#### β-galactosidase

Infections with βgal-AcMNPV were carried out in the same manner as that for studies of WT AcMNPV virus yields (as described above). At each time point, infected cells were collected by gently scraping cells from plates and pelleting by low speed centrifugation (700 × g, 5 min). The cell pellet was rinsed once with 1 ml phosphate-buffered saline (pH 7.0), then lysed in 200 μl of lysis buffer (20 mM Tris-HCl, pH 7.5, 5 mM EDTA, 150 mM NaCl, 1% Triton X-100). Debris were removed by centrifugation (12,000 × g for 5 min) and 2 μl of the supernatant was then added to 80 μl of Z buffer (60 mM Na_2_HPO_4_, 40 mM NaH_2_PO_4_, 10 mM KCl, 1 mM MgSO_4_, 50 mM β-mercaptoethanol, pH 7.4) in a 96 well plate. Twenty μl of pre-warmed substrate solution (OPNG, 4 mg/ml in Z buffer) was added to each well and absorbance at 420 nm was monitored every 1 min for 15 min. β-galactosidase activity was calculated according to the equation: U/ml = (OD_420 _· 0.102 ml · dilution factor)/(0.0045 [extinction coefficient] · sample volume).

### SEAP

Infection with SEAP-AcMNPV was carried out in a same manner as that for studies of WT AcMNPV virus yields, as described above. Endogenous alkaline phosphatase activity present in the infected culture medium was inactivated by incubation of supernatant samples at 65°C for 5 min. Two μl of each infected-cell supernatant sample (containing SEAP) was mixed with 198 μl of SEAP assay buffer (1.0 M diethanolamine, pH 9.8, 0.5 mM MgCl_2_, 10 mM homoarginine) in a well of a 96 well plate, and the mixture was incubated at 27°C for 30 min. Twenty μl of freshly prepared and pre-warmed substrate solution (120 mM p-nitrophenylphosphate in SEAP assay buffer) was added to each well. Absorbance at 405 nm was monitored every 1 min for 15 min. SEAP production (units/ml) was calculated according to the equation: U/ml = (change in absorbance × 220 μl × dilution factor)/(sample volume × 18.8 [extinction coefficient] × 1 cm).

### Analysis of N-glycan processing

Recombinant SEAP from the culture medium of Sf-900III-adapted Ao38 cells infected with SEAP-AcMNPV (MOI 10) was collected at 96 hr p.i. The culture medium was cleared by centrifugation at 2,000 × g for 10 min. The culture medium of WT AcMNPV-infected cells was prepared in the same manner and used as a SEAP-negative control. Proteins from each culture medium were separated on 8% polyacrylamide gels by SDS-polyacrylamide gel electrophoresis and visualized by Coomassie brilliant blue staining. Because the BV GP64 protein has a similar migration with SEAP, supernatant samples were also subjected to ultracentrifugation at 80,520 × g for 60 min to pellet BV particles, as a control. GP64 did not appear to be detectable in supernatant samples prepared as described above. Bovine serum albumin protein (0.1 to 2.0 μg) was electrophoresed in parallel to generate a standard curve for quantifying SEAP in the stained SDS-PAGE gels. The identity of the SEAP band was also confirmed by in-gel alkaline phosphatase assay after in-gel protein re-folding [29]. Briefly, after the SDS-polyacrylamide gel electrophoresis, proteins were incubated in a transfer buffer (0.01% Triton X-100, 48 mM Tris, 39 mM glycine, 20% methanol, pH 9.2) twice for 15 min. The gel was rinsed in a Western blot detection buffer for 5 min and alkaline phosphatase activity was detected by staining with BCIP/NBT. A band containing approximately 1 μg of the SEAP protein was excised from the gel and subjected to in-gel digestion and tryptic peptide extractions following a previously published protocol [30]. Briefly, gel slices were destained, reduced with 10 mM DTT and alkylated by treatment with 55 mM iodoacetamide in 20 mM ammonium bicarbonate, (in the dark at room temperature for 1 hr). Samples (gel slices) were incubated overnight in 40 μl of 25 mM ammonium bicarbonate containing 0.2 μg trypsin. The resulting tryptic-digested peptides in the solution were collected after centrifugation for 2 min at 4,000 × g. The remaining peptides in the gel were then extracted by sonication in 50 μl of 5% formic acid in 50% acetonitrile and collected similarly. All collected tryptic peptides were combined and evaporated to dryness in a Speedvac SC110 (Thermo Savant, Milford, MA). The digested samples were reconstituted in 15 μl of 2% acetonitrile with 0.5% formic acid, of which 4 μl were injected using a Famous auto sampler onto a C18 column (5 μm, 300 μm × 5 mm, Dionex) for on-line desalting and then separated on a PepMap C-18 RP nano column (3 μm, 75 μm × 15 cm, Dionex), eluted in a 45-minute gradient of 5% to 45% acetonitrile in 0.1% formic acid at 275 nl/min. The nanoLC was connected in-line to a hybrid triple quadrupole linear ion trap mass spectrometer, 4000 Q Trap equipped with a Micro Ion Spray Head II ion source (Applied Biosystems/MDS SCIEX, Framingham, MA).

MS data acquisition was performed using Analyst 1.4.2 software (Applied Biosystems) for precursor ion scan triggered information dependent acquisition (IDA) analysis and enhanced MS-based IDA analysis. The precursor ion scan at m/z 204.08 was monitored at a step size of 0.2 Da across a mass range of m/z 400 to 1500 for detecting glycopeptides containing N-acetylhexoamine unit. The nanospray voltage was 2.0 kV, and was used in positive ion mode for all experiments. The declustering potential was set at 50 eV and nitrogen as collision gas. In IDA analysis, after each precursor ion scan or EMS scan, and enhanced resolution scan, the two to three highest intensity ions with multiple charge states were selected for tandem MS (MS/MS) with rolling collision energy applied for detected ions based on different charge states and m/z values. All acquired MS/MS spectra from EMS-IDA were subjected to Mascot database search against Human database. All acquired MS/MS spectra for detected glycopeptides ions by precursor ion scanning were manually inspected and interpreted with BioAnalysis 1.4 software (Applied Biosystems). The peak areas of detected precursor ions were determined by extracted chromatogram (XIC) at each specific m/z representing glycopeptides isoforms. The relative quantitations of the glycan isoforms of N-linked peptide ions were carried out based on precursor ion peak areas under the assumption that all glycan isoforms linked to the same core peptide have identical or similar ionization efficiency.

### RT-PCR analysis of various cell lines for TNCL Virus

Ao, Sf9 and High Five cell lines were examined for the presence of an alphanodavirus (Tn5 cell line virus or TNCL Virus), which was recently shown to persistently infect cultures of High Five cells. TNCL Virus is produced in High Five cells at a significant level when challenged by wild type or recombinant AcMNPV [13]. To detect TNCL Virus, five sets of primers, which cover the bipartite TNCL genome segments (RNA1 and 2) were designed (Table 2) and used for one-step RT-PCR analysis of total RNA from Ao38, AoP (passage 5), High Five (passage 98) and Sf9 cells that were all infected with WT AcMNPV at 10 IU/cell. Total RNA was extracted from the infected cells at 48 hr p.i. using Trizol reagent (Invitrogen) according to the manufacturer's protocol. Each RT-PCR reaction (25 μl) consisted of 100 ng total RNA, 0.5 μM of each primer, 0.25 μl of SuperScript III RT/Platinum Taq mix, 1 × reaction mix (0.2 mM of each dNTP, 1.6 mM MgSO_4_). RT-PCR was run under the following conditions: 1 cycle of 45°C, 30 min; 1 cycle of 94°C, 2 min; 33 cycles of 94°C, 30 sec/55°C, 15 sec/68°C, 45 sec; 1 cycle of 68°C, 5 min. RT-PCR products were separated on a 1.5% agarose gel.

A portion of the total RNA sample (5 μg) was treated with DNase I (2 units) in a 50 μl reaction containing 1 × DNase I reaction buffer (10 mM Tris-HCl, pH 7.6, 2.5 mM MgCl_2_, 0.5 mM CaCl_2_) at 37°C for 30 min, and the reaction was stopped by adding 1 μl of 0.5 M EDTA followed by heating the mixture at 75°C for 10 min. Mock DNase I treated samples were prepared and processed in parallel by replacing DNase I with water. One hundred ng of DNase I-treated total RNA was used in PCR and one-step RT-PCR using AcMNPV *gp64 *primers (Table 2). Each PCR reaction (25 μl) consisted of 0.625 unit of Go-Taq DNA polymerase, 1.5 mM MgCl_2_, 0.2 mM each of dNTP, 0.2 μm each primer, 1 × Colorless Go-Taq Reaction buffer (Promega). PCR was run under the following conditions: 1 cycle of 95°C, 2 min; 33 cycles of 95°C, 30 sec/55°C, 30 sec/72°C, 30 sec; 1 cycle of 72°C, 5 min and the PCR products were separated on a 1.5% agarose gel.

## Authors' contributions

GWB conceived the initial project. YH developed methods and designed experiments for cell line generation. GWB and YH designed experiments on cell line characterization, virus infectivity, and recombinant protein production. YH performed those experiments and GWB and YH analyzed the data and wrote the manuscript. SZ, YH, and GWB designed glycan analysis studies. SZ performed the experiments, analyzed glycan data, and wrote a portion of the manuscript. All authors edited, read and approved the final manuscript.

## Supplementary Material

Additional file 1 **Table S1**. Proposed structures of the N-glycans born by SEAP glycopeptides observed by LC- MS/MS analysis of SEAP expressed by Ao38 cells Click here for file
