# Correction: BTI-Tnao38, a new cell line derived from *Trichoplusia ni*, is permissive for AcMNPV infection and produces high levels of recombinant proteins

**DOI:** 10.1186/1472-6750-12-12

**Published:** 2012-04-24

**Authors:** Yoshi Hashimoto, Sheng Zhang, Shiying Zhang, Yun-Ru Chen, Gary W Blissard

**Affiliations:** 1 Boyce Thompson Institute at Cornell University, Tower Road, Ithaca, NY 14853, USA; 2 Proteomics and Mass Spectrometry Facility, Cornell University, Tower Road, Ithaca, NY 14853, USA

## Abstract

After publication we discovered an error in the identification of the origin of the cell line reported in our article in BMC Biotechnology (2010, 10:50), entitled "Ao38, a new cell line from eggs of the black witch moth, *Ascalapha odorata *(Lepidoptera: Noctuidae), is permissive for AcMNPV infection and produces high levels of recombinant proteins". Upon analysis of primary *A. odorata *cultures, we found that they were contaminated with cells of *Trichoplusia ni *origin. The origin of the Ao38 cell line was determined as *T. ni *using three marker genes and the Ao38 cell line was renamed BTI-Tnao38. References to the origin of the cell line as *Ascalapha odorata *should be replaced with "a cell line of *Trichoplusia ni *origin". The absence of TNCL virus detection in the BTI-Tnao38 (Ao38) cell line was confirmed using a highly sensitive RT-PCR protocol capable of detecting TNCL virus RNA at approximately 0.018 copies/cell. Because of these observations, we have revised the title of the original article to "Correction: BTI-Tnao38, a new cell line derived from *Trichoplusia ni*, is permissive for AcMNPV infection and produces high levels of recombinant proteins" and two additional authors were added to reflect their contributions to the analysis of this cell line.

## Correction

After publication of this work [1], we discovered an error in the identification of the origin of the cell line reported in this study. In this study, we described the isolation and detailed characterization of a cell line that produces high levels of recombinant proteins. In addition, we were unable to detect the presence of the *Trichoplusia ni *cell line virus (TNCLV), an alphanodavirus that was previously reported in a *T. ni *derived cell line [2]. We reported that the Ao38 cell line was derived from a culture of primary cells of *Ascalapha odorata*. In the course of our subsequent characterization, we discovered that the source of the Ao38 cell line was not *A. odorata *as reported, but was instead *T. ni*. A careful analysis of archived intermediate stages in the production of the cell line indicated that the line resulted from primary *A. odorata *cultures that were contaminated with *T. ni *cells. While the source of the contamination is not known with certainty, further analysis suggests that Ao38 cells are likely a clonal derivative of the High Five (Tn-5B1-4) cell line. While we stand behind the analysis of the characteristics of this cell line, we are providing a correction to the above study, and we sincerely apologize for any problems or difficulties caused by our error in identification of the origin of this line. Below, we provide representative data establishing the identity of this line as a *T. ni *derived cell line. We also reexamined the cell line for the presence of TNCLV under conditions of defined sensitivity, and provide additional data confirming the absence of detection of the TNCLV alphanodavirus from this line.

To identify the species of origin of the Ao38 cell line, we amplified sequences from three marker genes that have been used to distinguish between and within lepidopteran species: a) mitochondrial cytochrome c oxidase subunit 1 (COI) [3-5], b) internal transcribed spacers (ITS) of the nuclear ribosomal 18S-5.8S-28S cistron [6-8], and c) a *cadherin *gene [9] (Figure 1). After PCR amplification and sequencing of DNA from selected regions of the COI gene and ribosomal ITS region, we found identical alignment of sequences from Ao38 cells and High Five (*T. ni*) cells, and nearly identical alignment with sequences from *T. ni *larvae (Figure 1A and 1B), as well as with *T. ni *sequences in the BOLD database [3,4]. Control sequences amplified from Sf9 cells and an *A. odorata *adult moth showed substantial variation (Figure 1A and 1B, Sf9 and *A. odorata*). Thus, the *T. ni *origin of the Ao38 cells was established using these sensitive marker genes. To examine the origin of the Ao38 cell line within the species *T. ni*, we examined *cadherin *gene sequences. A fragment of the *T. ni cadherin *gene sequence that contains an intron, was previously found to be highly polymorphic in sequence [9]. Therefore, we amplified and compared those *cadherin *sequences from several sources of *T. ni*. The target *cadherin *sequence from Ao38 cells was identical to that from High Five cells but differed from that of other *T. ni *sources. Figure 1C shows 100% identity between High Five and Ao38 sequences over a 415 bp region of the *cadherin *sequence. In contrast, sequences derived from larvae of a laboratory strain of *T. ni *(Cornell strain [10]), and from a recently reported *T. ni *cell line (QB-9-4s) [11], showed app. 2.4% sequence divergence over the same region. Thus, the Ao38 line appears to be closely related to, and is likely derived from High Five cells. Based on these data, we decided to rename the Ao38 cell line as BTI-Tnao38.

**Figure 1:**
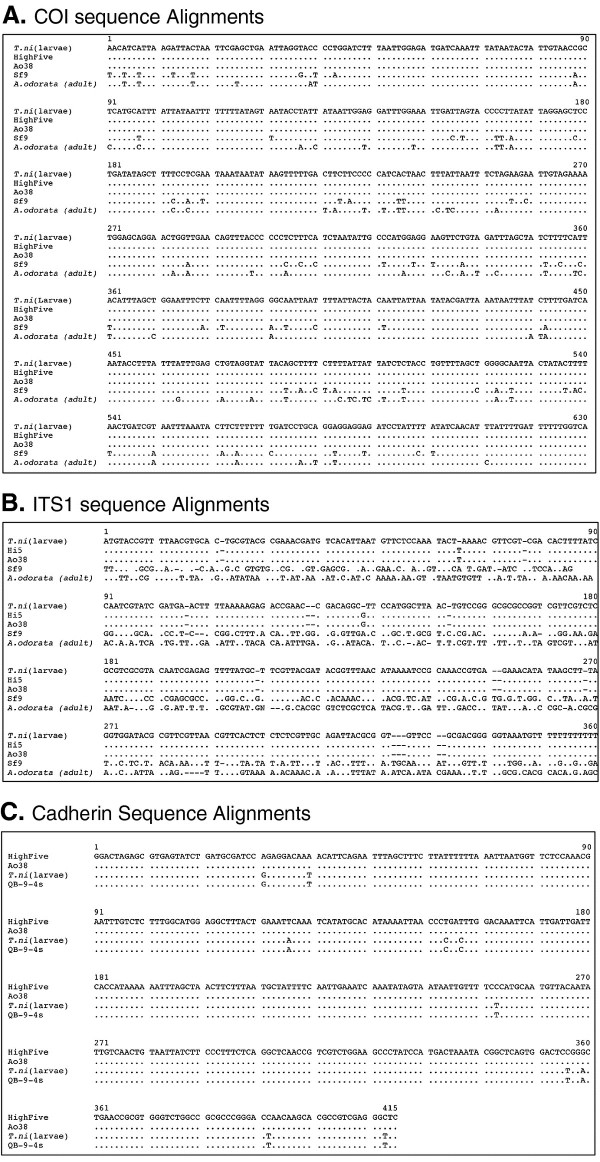
**Alignments of marker gene sequences derived from Ao38 cells, and those from reference cell lines and insects**. **A**. Alignment of DNA sequences PCR amplified from the mitochondrial cytochrome oxidase c subunit 1 (COI) gene. Primer pairs LCO1490 (5'-GGTCAACAAATCATAAAGATATTGG-3') and HCO2198 (5'-TAAATCTCAGGGTGACCAAAAAATCA-3') [12] were used for PCR amplification of an approximately 658 bp fragment using a previously described method [5] with minor modifications. Reference DNA samples used for comparisons were derived from *T. ni *larvae (Cornell strain; kindly provided by Dr. Ping Wang), High Five (Tn-5B1-4) cells, Sf9 cells, and an *Ascalapha odorata *adult. Species identities were also confirmed by analysis using the BOLD database [3,4,13]. For all alignments, "." represents nt sequence identity, and differences are indicated by nt abbreviations (A, C, G, or T). **B**. Alignment of sequences amplified from the internal transcribed spacers (ITS) of the nuclear ribosomal 18S-5.8S-28S cistron. Primer pairs ITS1-1 (5'-CCCCATAAACGAGGAATTCC-3') and ITS4 (5'-TCCTCCGCTTATTGATATGC-3') [8] were used for PCR amplification. An amplicon with a size of approximately 1500 bp was amplified. Reference DNA samples were the same as those used for analysis of COI (above). **C**. Alignment of cadherin fragment sequences from *T. ni *larvae and cell lines. The cadherin fragment sequences were PCR amplified from the indicated sources using PCR primers PW-206 (5'-CGCTTTGATGGTCTCGTTC-3') and PW-261 (5'-GCGCTGCTGGGCTTCCTGT-3') as described previously [9]. A 415 nt sequence alignment is shown. Sequences from two *T. ni *cell lines (High Five and QB-9-4s) and a *T. ni *larval sample (Cornell strain, provided by Ping Wang) are aligned with the sequence from Ao38 cells.

The alphanodavirus TNCLV is readily detected by RT-PCR in growing High Five cells. We previously reported that we were unable to detect TNCLV in the Ao38 cell line by RT-PCR. We therefore performed a careful reexamination of the Ao38 cells for the presence of TNCLV RNA, and measured the sensitivity of our methods. For these studies, we amplified and cloned a 566 nt region of the TNCLV genome, then *in vitro *transcribed and purified an RNA representing that region of the TNCLV genome. The *in vitro *transcribed TNCLV RNA was subsequently used for spiking experiments with Sf9 cell RNA, to determine the sensitivity of TNCLV RNA detection in our RT-PCR studies (Figure 2). Under these conditions we found that the sensitivity for detection of TNCLV RNA was approximately 53 molecules of TNCLV RNA in the background of total RNA from approximately 3000 Sf9 cells (Figure 2, lanes 3-7). Under these conditions, TNCLV was not detected in RNA isolated from approximately 3000 cells of the Ao38 line (Figure 2, lane 8). In a parallel analysis, Sf9 cells were also negative for TNCLV, as expected (lane 2). Thus, based on this level of sensitivity, we estimate that if TNCLV is present in Ao38 or Sf9 cells, it would be found at less than one TNCLV genome per 50 cells. In contrast to Ao38 and Sf9 cells, TNCLV RNA was detected at relatively high levels in High Five cells. Based on the sensitivity of detection in our TNCLV RNA spiking experiment, and the levels of TNCLV RNA detected in dilutions of High Five cell RNA (Figure 2, lanes 9-11), we estimate that TNCLV RNA is present at levels of > 180-300 copies per cell in High Five cells. The marker genes and methods described above may be useful for confirmation or validation of cell lines, and the methods used for alphanodavirus detection may also be useful for laboratories examining *T. ni *or other cell lines suspected to harbor this alphanodavirus.

**Figure 2:**
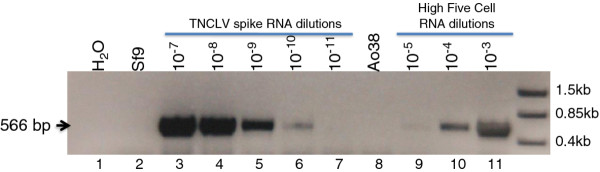
**RT-PCR Analysis of cell line RNA for detection of the alphanodavirus, TNCLV**. To determine the sensitivity of TNCLV RNA detection, a segment of TNCLV RNA 1 (2368-2933 nt) was synthesized *in vitro *and used to spike RNA from Sf9 cells. 250 ng of the *in vitro *RNA fragment was used for a series of serial dilutions (10^-1 ^to 10^-12^) and each diluted RNA fragment sample was added to 250 ng of Sf9 total cell RNA (representing approximately 3000 cells). One-step RT-PCR was performed using the Invitrogen SuperScript III One-Step RT-PCR System with Platinum Taq DNA Polymerase under the following conditions: 30 min at 45°C, and 94°C for 2 min for 1 cycle; followed by 94°C for 15 sec, 55°C for 30 sec, and 72°C for 45 sec for 40 cycles; then 10 min at 72°C. The sensitivity of detection was estimated as approximately 1 TNCLV RNA per 50 cells. Ao38, High Five and Sf9 cell RNAs were examined for TNCLV using the same primers and conditions for RT-PCR as described for spiking experiments. For High Five cells, total cellular RNA (250 ng; representing an estimated 3000 cells) was diluted as indicated (10^-3 ^to 10^-5^). For analysis of Ao38 and Sf9 cells, 250 ng of cell RNA was analyzed directly by RT-PCR as described above.

To reflect the significant contributions of scientists involved in the extended analysis of the origin of the Ao38 cell line, and the analysis of TNCLV RNA detection and sensitivity, Shiying Zhang and Yun-Ru Chen were included as authors of this study.

## Authors' contributions

GWB conceived the initial project. YH developed methods and designed and performed experiments for cell line generation. GWB and YH designed experiments on cell line characterization, virus infectivity, and recombinant protein production. YH performed those experiments and GWB and YH analyzed the data and wrote the manuscript. Sheng Z, YH, and GWB designed glycan analysis studies. Sheng Z performed the experiments, analyzed glycan data, and wrote a portion of the manuscript. Sheng Z, YH, and GWB edited, read and approved the original manuscript. YC and Shiying Z designed experiments and performed analysis of species-specific marker genes. Shiying Z and GWB designed experiments on alphanodavirus detection and Shiying Z performed the experiments and analyzed the data. GWB wrote the correction and all authors edited, read and approved the correction.
