# Supplementary material for: Ao38, a new cell line from eggs of the black witch moth, Ascalapha odorata (Lepidoptera: Noctuidae), is permissive for AcMNPV infection and produces high levels of recombinant proteins
Source: BMC Biotechnol. 2010 Jul 5;10:50. doi: 10.1186/1472-6750-10-50 (PMC2906426; doi:10.1186/1472-6750-10-50)
Supplement: Additional file 1 — Table S1. Proposed structures of the N-glycans born by SEAP glycopeptides observed by LC- MS/MS analysis of SEAP expressed by Ao38 cells [file 1472-6750-10-50-S1.PDF]

Table S1 Proposed structures of the N-glycans born by SEAP glycopeptides observed by LC-MS/MS analysis of SEAP expressed by Ao38 cells

Glyco-peptides M+H	Ret min	m/z	z	M+H	Sugar	Peak area	Relative %#	Possible glycan structure*	Glycan
FNQCN[139]TTR C is carbamidomethyl-cysteine M+H=1040.4578	8.46	959.2	2	1917.4	876.8	2.35E+04	24.28	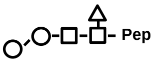	Man2Gn2F1
	8.46	1040.1	2	2079.2	1038.7	5.08E+04	52.48	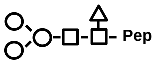	Man3Gn2F1
	8.46	1121.0	2	2241.0	1200.5	1.65E+04	17.05	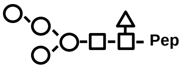	Man4Gn2F1
	8.46	1113.1	2	2225.2	1184.5	6.00E+03	6.20	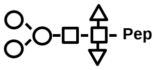	Man3Gn2F2
			Sum			9.68E+04	100.00		
FNQCN[139]TTR M+H=1054.4737 C is carbamidomethyl-homocysteine or Propionamide-cysteine	8.69	966.2	2	1931.4	876.8	7.70E+04	34.76	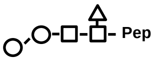	Man2Gn2F1
	8.69	1047.1	2	2093.2	1038.7	1.31E+05	59.27	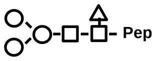	Man3Gn2F1
	8.69	1128.0	2	2255.0	1290.5	7.81E+03	3.53	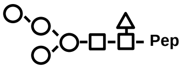	Man4Gn2F1
	8.69	1120.1	2	2239.2	1184.5	5.40E+03	2.44	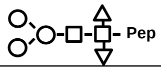	Man3Gn2F2
			Sum			2.22E+05	100.00		
YVWN[266]R M+H=737.3729	20.05	977.5	2	1954	1216.8	1.71E+05	13.39	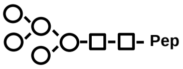	Man5Gn2
	20.05	1058.6	2	2116	1378.8	4.00E+05	31.33	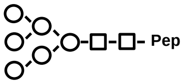	Man6Gn2
	20.7	815.5	2	1631	892.8	3.74E+05	29.29	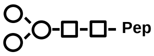	Man3Gn2
	20.6	896.5	2	1792	1054.8	2.86E+04	2.24	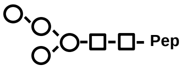	Man4Gn2
	20.6	917	2	1834	1096	5.23E+04	4.10	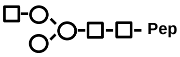	GnMan3Gn2
	21.2	734.4	2	1469	731	2.51E+05	19.66	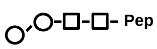	Man2Gn2
			Sum			1.28E+06	100.00		

*, Circle, mannose; square, N-acetylglucosamine; triangle, fucose

#, Based on the assumption that all glyco-isoforms for the same core peptide share a similar ionization efficiency
